# Rapid Kill—Novel Endodontic Sealer and *Enterococcus faecalis*

**DOI:** 10.1371/journal.pone.0078586

**Published:** 2013-11-06

**Authors:** Nurit Beyth, Dana Kesler Shvero, Nathan Zaltsman, Yael Houri-Haddad, Itzhak Abramovitz, Michael Perez Davidi, Ervin I. Weiss

**Affiliations:** 1 Department of Prosthodontics, Faculty of Dentistry, The Hebrew University-Hadassah, Jerusalem, Israel; 2 Department of Endodontics, Faculty of Dentistry, The Hebrew University-Hadassah Jerusalem, Israel; Universidad Nacional de La Plata., Argentina

## Abstract

With growing concern over bacterial resistance, the identification of new antimicrobial means is paramount. In the oral cavity microorganisms are essential to the development of periradicular diseases and are the major causative factors associated with endodontic treatment failure. As quaternary ammonium compounds have the ability to kill a wide array of bacteria through electrostatic interactions with multiple anionic targets on the bacterial surface, it is likely that they can overcome bacterial resistance. Melding these ideas, we investigated the potency of a novel endodontic sealer in limiting *Enterococcus faecalis* growth. We used a polyethyleneimine scaffold to synthesize nano-sized particles, optimized for incorporation into an epoxy-based endodontic sealer. The novel endodontic sealer was tested for its antimicrobial efficacy and evaluated for biocompatibility and physical eligibility. Our results show that the novel sealer foundation affixes the nanoparticles, achieving surface bactericidal properties, but at the same time impeding nanoparticle penetration into eukaryotic cells and thereby mitigating a possible toxic effect. Moreover, adequate physical properties are maintained. The nanosized quaternary amine particles interact within minutes with bacteria, triggering cell death across wide pH values. Throughout this study we demonstrate a new antibacterial perspective for endodontic sealers; a novel antibacterial, effective and safe antimicrobial means.

## Introduction

It is well recognized that biofilms respond poorly to conventional antibiotics and may develop resistance to antibiotics. Moreover, the widespread use of antibiotics leads to the emergence of more resistant and virulent strains of microorganisms. Consequently, the detection of new antimicrobial means is becoming of major importance for novel treatment options [1]. In the oral cavity, failure of root canal (endodontic) treatment is often caused by the persistence of microorganisms in the root canal system after therapy, or recontamination of the root canal owing to inadequate sealing. Furthermore, microorganisms are essential to the development of periradicular diseases and are the major causative factors associated with endodontic treatment failure [2]. Retreatment calls for removal of the original root canal filling, further instrumentation, disinfection, and refilling [3]. Ideally, for optimal endodontic treatment outcome, the bacterial population within the root canal should be eliminated or at least significantly reduced to levels that are compatible with periradicular tissue healing. If bacteria remain after chemo-mechanical preparation, with or without intracanal medication, there is an increased risk of an adverse outcome of the endodontic treatment. Indeed, the bacterial presence in the root canal at the time of filling has been shown to be a risk factor for post treatment apical periodontitis [4]. Intracanal infection may persist owing to anatomical limitations that are not favorable to chemo-mechanical preparation [5], ineffective irrigation [6], and ineffective mechanical preparation that leave about a third of the root surfaces untouched [7]. A common resistant intracanal pathogen that serves as a gold standard bacterium in endodontic research is *Enterococcus faecalis* (*E. faecalis*) [8]. *E. faecalis* is a highly resistant pathogen, demonstrating resistance to various irrigates and medicaments, such as sodium hypochlorite [8], and the most popular medication, Ca(OH)_2_ [9]. Coronal leakage may also cause failure of endodontic therapy [10], [11]. As these areas have intimate contact with the root canal sealing material [12], it would be beneficial if sealing materials retained their antibacterial properties. Regrettably, most endodontic sealing materials (endodontic sealers) are devoid of antibacterial properties; the few that were found to have an antibacterial effect lose this quality within a week [13]–[16]. Moreover, endodontic microorganisms have been shown to have a high affinity for root canal filling materials, especially for gutta-percha. Because of the substantial bacterial adhesion, subsequent biofilm formation can lead to the persistency of the microorganisms in the root canals [17].

The use of insoluble polycationic disinfectants as a non-release strategy for creating bactericidal surfaces is gaining interest in modern medicine. Antimicrobial polycations include ion exchange fibers [18], alkoxysilanes [19], insoluble pyridinium-type polymers [20], [21], polyionenes [22], polymer surface derivatives with poly(vinyl-N-pyridinium) [22]–[24] and immobilized N-alkylated polyethyleneimine (PEI) [25]–[27]. It is generally accepted that polycationic disinfectants exhibit a broad-spectrum antimicrobial activity. Whereas conventional antibiotics are becoming less effective in the treatment of biofilm infections, bacteria do not appear to develop resistance to cationic polymers because of their non-specific mode of action [24], [26], [28]. It was previously hypothesized that the positive surface charge and hydrophobic moieties of the polycationic disinfectants allow an electrostatic interaction, followed by penetration through the cell wall and resulting in cell membrane leakage of intracellular contents and cell death [29]. In particular, it was reported that quaternary ammonium polyethyleneimine (QPEI) possesses excellent antibacterial activity [30]. When these polymers were synthesized as insoluble antibacterial nanoparticles that were incorporated into resin-based materials, potent and long-lasting antibacterial surface properties were attained both *in vitro* [30]–[32] and *in vivo* [33]. The antibacterial compound is stable and does not leach out from the material into the surrounding environment, rendering perpetual antibacterial surface properties. Moreover, although bactericidal-immobilized materials usually display an inactivating effect only against bacteria that come in contact with the antibacterial molecules, when QPEI nanoparticles were incorporated in resin-based materials, bacterial death was induced in *vivo* even in the outermost parts of the biofilm [33].

In the present study, a novel endodontic sealer is described. A method for fabricating optimized nanoparticles adjusted to epoxy-based materials, providing a well-dispersed endodontic sealing material incorporating QPEI nanoparticles is demonstrated. Adjustment of the QPEI nanoparticles provides an extensive reacting surface, allowing stable affixation to the epoxy-based sealer matrix. We hypothesized that the novel endodontic sealer has a potent antibacterial effect on endodontic pathogenic bacteria, and examined the sealing material for its antibacterial efficacy, biocompatibility, physical and chemical properties.

## Materials and Methods

### Test materials

**QPEI nanoparticle preparation.** Synthesis was as previously described [30]. Briefly, nanosized particles were prepared by dissolving PEI in ethanol that was reacted with dibromopentane under reflux for 24 hrs. N-alkylation was conducted using octyl. Alkylation was carried out under reflux for 48 hrs followed by 24 hrs neutralization with sodium bicarbonate. Then N-methylation, using methyl iodide, was conducted at 42°C for 48 hrs followed by 24 hrs neutralization with sodium bicarbonate. The supernatant obtained was decanted and precipitated in double distilled water (DDW), washed with hexane and DDW and then freeze-dried. The average yield was ≥ 85% (mol/mol). Then the particles were washed with a 2% solution of N-lauryl-sarcosine surfactant (NLS). Prepared QPEI nanoparticles (20 g) were placed in a Buchner funnel using a paper filter and a vacuum source. A volume of 200 ml of NLS solution was passed through the nanoparticles under vacuum conditions. The treated nanoparticles were freeze-dried overnight to obtain a fine powder. Fourier transform InfraRed (FTIR) spectra were recorded on a Perkin Elmer, 2000 FTIR. QPEI nanoparticle FT-IR: 3,424 cm-1 (N–H), 2,954, 2,924 and 2,854 cm-1 (C–H), 1,617 cm-1 (N–H, small band), 1,465 cm-1 (C–H), 917 cm-1 quaternary nitrogen. Average particle size (31.52 nm) was recorded using a size analyzer (Zetasizer nano ZS, Malvern).

RCS (B.J.M Laboratories Ltd, Or-Yehuda, Israel), a two-paste epoxy-amine resin endodontic sealer, was used (composition: paste 1- bisphenol-A- (epichlorhydrin) and epoxy resin, zirconium oxide, silica, iron oxide pigments; paste 2- poly - oxyethylene, oxypropylene, diamine, silicon oil, silica, zirconium oxide). QPEI nanoparticles were added to paste 2 at 0% or 1.5% (wt/wt). RCS with or without nanoparticles was mixed using a dual auto-mixing syringe according to the manufacturer's instructions.

### Antibacterial properties

The antibacterial efficacy of the QPEI nanoparticles was tested against *E. faecalis* a common, resistant intracanal pathogen [8]. To substantiate the nanoparticles' antibacterial effect, they were tested when immobilized (novel endodontic sealer). To establish the mode of action, the nanoparticles’ effect was further evaluated in suspension.

**Preparation of bacterial suspension.** *E. faecalis* (isolated at the Maurice and Gabriela Goldschleger School of Dental Medicine, Tel Aviv University, Israel), was cultured overnight in 5 mL of brain-heart infusion broth (BHI) (Difco, Detroit, MI, USA), at 37°C under aerobic conditions. The top 4 mL were transferred to a fresh test tube and centrifuged for 10 min at 4,165 x g. The supernatant was discarded and the bacteria were resuspended in 5 mL of PBS and vortexed gently for 10 sec.

**Antibacterial effect of immobilized nanoparticles.** The surface antimicrobial effect of the novel endodontic sealer RCS, incorporating 0% or 1.5% (wt/wt) QPEI nanoparticles, was tested. A microtiter plate (96-well flat bottom plate Nunclon, Nunc, Copenhagen, Denmark), was positioned vertically and the sidewalls of 8 wells were coated with similar amounts of the tested material, mixed according to the manufacturer’s instructions. Triplicate microtiter plates were similarly prepared and tested after 1 month of material aging. During this time, each well was filled with 250 µl phosphate-buffered-saline (PBS; Sigma, St. Louis, MO. USA), which was replaced every 48 hrs, and the plates were incubated at 37°C. The plates were dried before testing. The antibacterial effect was determined using the direct contact test (DCT) [34]. A 10 µl volume of bacterial suspension (10^8^ CFU/mL) was placed on the surface of each tested material, and the plate was incubated vertically for 1h at 37°C. During this period the suspension liquid evaporated and a thin layer of bacteria was obtained, ensuring direct contact between all the bacteria and the tested surface. Then the plate was positioned horizontally and 220µl of BHI broth were added to each well. The microtiter plate was placed in a temperature-controlled microplate spectrophotometer (VERSAmax, Molecular Devices Corporation, CA, USA; Thermoscientific Multiscan FC microplate photometer, Thermo Fisher Scientific, Finland), at 37°C with 5 sec vortex mixing before each reading. Bacterial growth was estimated by following changes in O.D. at 650 nm in each well every 20 min for 14 hrs. Controls included equal volumes of the bacterial suspension placed on the sidewalls of 8 polystyrene wells. Absorbance measurements were plotted, providing bacterial growth curves for each well in the microtiter plate, and the linear portion of the logarithmic growth phase was subjected to statistical analysis, the slope correlating with bacterial growth rate and the intercept correlating with total viable count. Calibration experiments were performed simultaneously in each plate. Triplicate wells containing 265 µl BHI were inoculated with 10 µl bacterial suspension. A fivefold dilution was repeated 7 times in triplicates. A gradual and reproducible decrease in O.D. correlated with serial dilution. The dilution at zero time resulted in a measurable delay in exponential growth phase. Growth curves obtained from each experimental well were superimposed on the calibration curves obtained, allowing the number of residual viable bacteria on each tested specimen at the end of the 1 hr incubation to be calculated. The results were analyzed using ANOVA followed by Tukey’s test (p<0.05). Each experimental set-up included viable cell counts (calculated as CFU/mL) of the control wells to validate that the absorbance measurements correlated with them. Scanning electron microscope (SEM) was used to visualize the *E. faecalis* cells during the 1 hr incubation period; i.e. after 20 and 40 min. RCS incorporating 0% or 1.5% nanoparticles were scanned. The samples were fixed with formaldehyde and gluteraldehyde and osmium tetroxide in cacodylate buffer, then dehydrated with a graded ethanol and Freon series, and coated with gold.

To examine the possible effect of leachable materials from the sealer materials, the antibacterial properties of the elute from the tested materials on planctonic growth was determined quantitatively. In a separate 96-well microtiter plate, eight sidewalls were coated with each tested material (RCS with or without nanoparticles) and aged for one month as described above. Then, a volume of 230 µl of BHI was added to each well and the plate was incubated for 24 hrs at 37°C. A 220 µl volume was transferred from each well to an adjacent set of wells and 10 µl of a bacterial inoculum, prepared as described above, were added to determine the effect of components eluted into the broth. The plate was placed in the temperature-controlled microplate spectrophotometer, set at 37°C, with 5 sec mixing before each reading. Bacterial growth was assessed by following the changes in O.D_._ (at A650 nm) every 20 min for 24 hrs. Growth curves were analyzed as described above in the DCT.

The antibacterial effect of the novel endodontic sealer was also tested using the agar diffusion test (ADT). Discs of RCS incorporating 0% or 1.5% (wt/wt) nanoparticles were prepared using a silicone mold (4.5 mm diameter; 3 mm height) and left to dry at 37°C. A suspension of *E. faecalis* (200 µl; 10^8^ CFU/ml) was spread on blood agar plates and the discs were placed on the surface. An antibiotic (5µg ciprofloxacin) disc served as control. The plates were incubated for 24 hrs at 37°C and the inhibition zones around each specimen were measured. The absence of an inhibition halo was scored zero. The inhibition diameter, including the disc diameter, was measured.

**Antibacterial effect of nanoparticles in suspension.** Tubes containing 9 ml sterile DDW and a QPEI nanoparticle (1 mg/mL) suspension were prepared, DDW tubes without nanoparticles served as control. A volume of 1 mL *E. faecalis* suspension (10^8^ colony forming units (CFU)/mL) was added to each tube followed by manual mixing. The tubes were incubated for 1 hr at 37 °C. Serial dilutions were made at consecutive time points, 0 min, 4 min, 8 min, 12 min, 30 min and 1 hr. Viable cell counts were calculated as CFU/mL. SEM samples were taken at each time point. The samples were fixed with formaldehyde and gluteraldehyde and osmium tetroxide in cacodylate buffer, then dehydrated with a graded ethanol and Freon series, and coated with gold. A High Resolution SEM, Sirion (Center for Nanoscience and Nanotechnology, The Hebrew University of Jerusalem, Israel) was used.

The antibacterial effect of the QPEI nanoparticles at various pH values was determined as similarly described by *Gao et al*. [29]. Sterile test tubes containing DDW were prepared. The pH value of each test tube was adjusted by adding dilute solutions of HCL (0.1 M) and NaOH (1 M) (9 mL total volume/tube). The QPEI nanoparticles were added to the test group tubes at 1 mg/mL. A 1 mL volume of the *E. faecalis* suspension (10^8^ CFU/mL) was added to the test and control tubes. The tubes were incubated at 37°C for 10 min with light vortexing every 3 min. Serial dilutions were made to determine viable cell counts, calculated as CFU/mL. The antibacterial ratio (growth inhibition) was calculated according to the following equation [29]:

Antibacterial ratio =  (No. of original cells- No. of viable cells)/ No. of original cells.

### Cytotoxicity assay

Cytotoxicity was tested based on ISO 10993 specifications. Three samples of each sealer were prepared according to the manufacturer’s instructions and stored in an incubator before UV sterilization. An agarose in water solution (3% (w/v) was autoclaved and cooled down in a water bath at 40°C for 1h before use. L929 growth medium was incubated in a water bath at 40°C for 36 min before use. Then the agarose solution was mixed with the heated L929 growth medium (1:1) and incubated at 40°C for 1 hr before use. Test products and controls were applied to the agarose layer, in indirect contact with the cells, based on the assumption that diffusible leachables might lead to cells damage or even cell lysis. This causes decolorization after staining with a viability indicator. After removal of the test materials, cytotoxicity was determined microscopically and the cells were stained with the indicator MTT that is metabolically reduced only in viable cells. The decolorization index and the lysis index were determined qualitatively, according to the size of the decolorized area and the number of lysed cells around and under the test products, respectively. Cell viability was graded as the decolorization index /lysis index. The positive and negative controls resulted in cell reactions that were scored: 2–3 - moderately to- severely cytotoxic and 0 – non-cytotoxic, validating the tests. Exponentially growing L929-W6-160910 cells were rinsed and detached from the flasks with 0.25% trypsin/EDTA solution. Trypsin activity was terminated by the addition of L929 growth medium and a single cell suspension was prepared. Individual wells of 6-well tissue culture plates were each filled with a 3 ml suspension of 2.5×10^5^ cells/ml L929 growth medium. The plates were incubated for 24 hrs; 37°C, humidified 5±0.5% CO_2_/air, to enable cell adherence, which was verified microscopically. The medium was then aspirated and 2.3 ml Agarose Overlay Solution, at 40°C, was carefully applied to the cell monolayer by dispensing down the side of the dish. To allow solidification, the plates were incubated at room temperature for 49 min. The cells were then incubated for 1h; 37°C, humidified 5±0.5% CO_2_/air. Following solidification of the agarose overlay solution; the test material (disc diameter, ∼ 5 mm) was applied directly to the agar. Three samples of each test material, the positive control and the negative control were applied in separate wells. The samples were kept in the center of the well, the plates were incubated at 37°C, humidified 5±0.5% CO_2_/air for 23.5 hrs. The following day the test materials were carefully removed from the cultures and the plates were observed microscopically for cell lysis. A 1.5 ml volume of MTT solution was applied to each well. After 2hrs incubation of the plates at 37°C a dark blue color was obtained. Excess MTT was removed. MTT-stained cultures were observed macroscopically for cell decolorization to determine the zone of cell lysis and the diameter of the lysed area was measured. Any other morphological changes were observed microscopically.

### Physical properties

Physical properties including solubility and the material flow of the novel endodontic sealer were tested. Additionally, thermal analysis was preformed. RCS incorporating 0%, 1%, 1.5% or 2% (wt/wt) QPEI nanoparticles were examined.

**Solubility.** Solubility tests were performed in accordance with ISO 6876:2001(E) specifications for root canal sealing materials.

A total 5 cylindrical polytetra-fluoroethylene molds (20 mm diameter; 1.5 mm height) were used for each test group. The molds were placed on glass slides covered with polyethylene film and filled with a slight excess of RCS. The smooth surface of the non-polymerized RCS was achieved by pressing a glass slide against the material’s surface. Samples were incubated at 37°C, >95% relative humidity for 36 hrs. Fully set samples were removed from the molds and weighed on an HM-200 precision scale (Mettler, Toledo**,** OH, USA) and the mean reading was recorded.

Samples were placed in a shallow glass dish with 50 ml distilled water, and placed in a 37°C chamber with >95% relative humidity for 1 week. The samples were then removed from the containers, rinsed with distilled and deionized water, and blotted dry with absorbent paper. The samples were then placed in a dehumidifier for 24 hrs and reweighed. The experiment was repeated five times. The weight loss of each sample, expressed as the percentage of the original mass, was taken as the solubility of the sealer. Minimal sample weight accuracy was 0.001 g.

**Thermal analysis.** Triplicate samples from each test group were prepared in 40 µl aluminum pans and allowed to polymerize for 24 hrs at 37°C. Thermal analysis of all samples was performed using a differential scanning calorimeter (DSC) device (Mettler). The samples were cooled to 0°C and then heated to 150°C at a heating rate of 10°C /min. Thermal transition values were calculated using STAR software. As the glass transition temperature (Tg) tends to appear as a broad thermal transition, only values for midpoint temperature are presented.

**Flow assay.** The flow test was conducted according to ISO 6876:2001(E) specifications. A volume of 0.05 mL (±0.005 mL) of the tested material was placed on a glass plate and covered with another plate placed centrally on top of the sealer, followed by a weight, giving a total mass of 120 g (±2 g). Ten minutes after initiating material mixing, the weight was removed and the maximum and minimum diameters of the compressed discs of the sealers were measured. Two conditions were necessary to validate the tests: the difference between the maximum and minimum diameters should not exceed 1.0 mm and the compressed discs should be of uniform shape. If these conditions were not met, the test was repeated. Three determinations were made and the mean value was calculated to the nearest millimeter. The minimal acceptable diameter was 20 mm.

## Results

### Antibacterial properties

**Figure 1:**
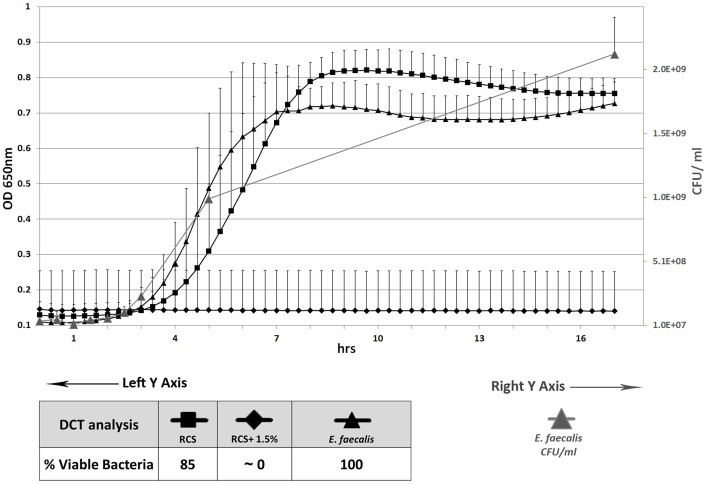
Antibacterial activity of novel endodontic sealer. Bacterial growth following direct contact with 4 weeks- aged RCS samples incorporating 0% or 1.5% (wt/wt) QPEI nanoparticles. Each point on the curve is the average absorbance (OD_650nm_) measured simultaneously in eight wells similarly prepared in the same microtiter plate. No bacterial growth was observed in the modified sealer. Data analysis showed total growth inhibition (6 log decrease in viable bacteria counts) in RCS samples incorporating QPEI nanoparticles (p<0.0001). RCS without nanoparticles showed <20% growth inhibition. Viable *E. faecalis* control count is shown as a superposed curve; right Y-axis, calculated as CFU/ml.

**Figure 2:**
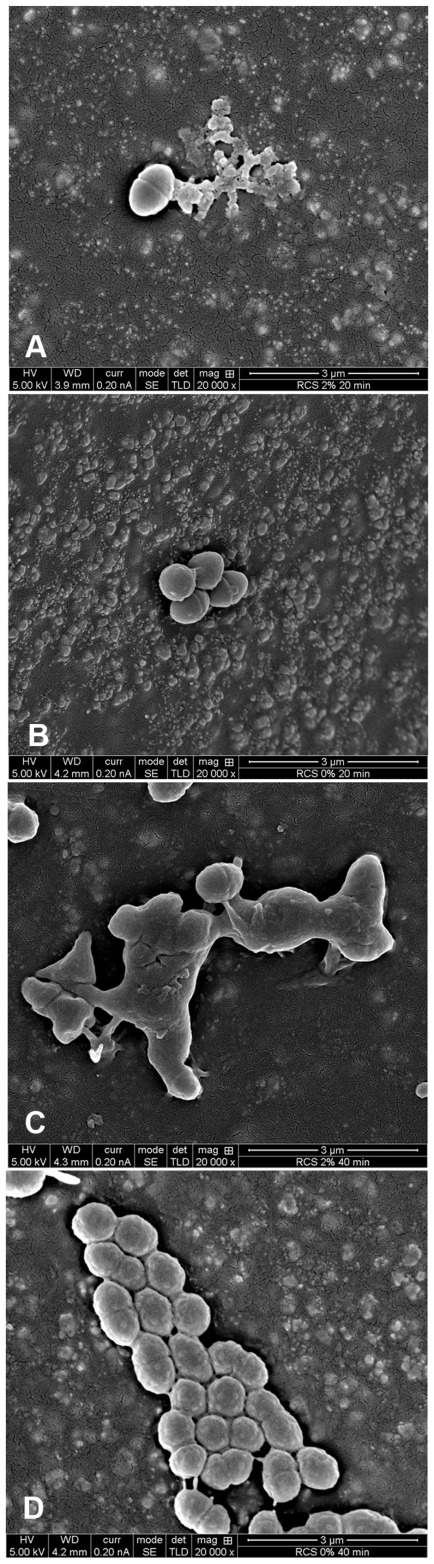
SEM images of *E. faecalis* following contact with the novel endodontic sealer and unmodified sealer. **A)** *E. faecalis* following 20 min direct contact with the surface of the novel endodontic sealer. Syncytium like cells and bacterial lysis can be observed. Most of the bacteria show changes in morphology with no visible signs of cell division. **B)** Bacteria following 20 min direct contact with a sealing material without QPEI nanoparticles, early biofilm formation with intact membrane and dividing cells can be observed. Bacteria on the surface of the novel sealer and the sealer without nanoparticles after 40 min, **C** and **D** respectively.

**Antibacterial effect of immobilized nanoparticles.** Total bacterial growth inhibition was observed in RCS samples incorporating 1.5% (wt/wt) QPEI nanoparticles (Fig. 1). Based on the linear portion of the logarithmic growth in the calibration curves, the number of residual viable bacteria on each tested specimen was determined and analyzed. In RCS samples with incorporated QPEI nanoparticles, the number of residual viable bacteria decreased 6 logs (p<0.0001) *vs.* the number in RCS without nanoparticles. Control group viable counts correlated with absorbance measurements. SEM images of the novel endodontic sealer and unmodified sealer following contact with *E. faecalis* revealed that most of the observed bacteria underwent changes in bacterial morphology and dispersion, with no visible signs of cell division (Fig. 2). Syncytium-like cells and bacterial lysis were observed after contact between the bacteria and a surface containing QPEI nanoparticles. In contrast, when the bacteria came in contact with the conventional sealing material, early biofilm formation with intact membranes and dividing cells was observed (Fig. 2).

The antibacterial properties of the eluted components released from the tested materials were evaluated. The *E. faecalis* growth curves were similar to those of the control group (Table 1). No inhibition halo was observed in the ADT in any of the experimental groups (Table 1).

**Table 1:** Antibacterial effect of eluted components on *E. faecalis*.

(%) nanoparticles in RCS	0	1.5
a **ADT**	**0**	**0**
b **Bacterial growth (elute)**	**100%**	**100%**

a Inhibition halo diameter (mm) measured by two perpendicular lines.

b Bacterial growth in the presence of soluble components eluted from the tested materials into the growth media expressed as percent control (control = 100%).

**Figure 3:**
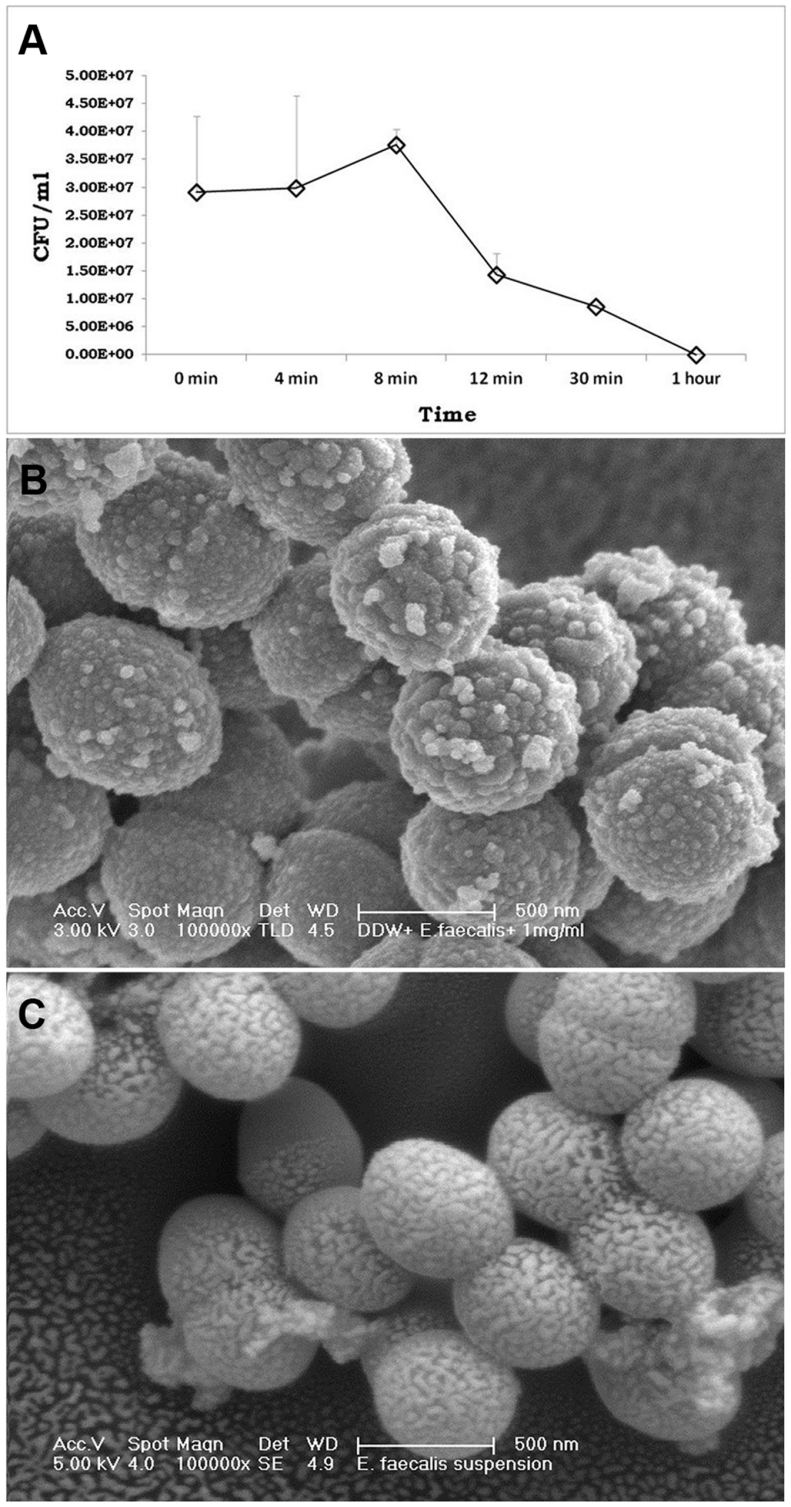
Determination of the antibacterial activity of QPEI nanoparticles in suspension. **A)** Viable counts of *E. faecalis* (CFU/ml) in DDW with QPEI nanoparticles determined in similarly prepared samples. Measurements were made at consecutive time points (0 min, 4 min, 8 min, 12 min, 30 min and 1 hr). A decrease in cell viability was recorded within 8 minutes. Scanning electron micrographs of *E. faecalis*. **B)** in DDW with QPEI nanoparticles- depicting attached nanoparticles on bacterial membranes; **C)** in DDW- depicting regular bacterial membranes.

**Antibacterial effect of nanoparticles in suspension.** Samples taken during the 1 hr incubation of *E. faecalis* inoculated in a QPEI nanoparticle suspension showed a decrease in viable counts after 12 mins, followed by a continuous decrease, with no viable bacteria at 1 hr (Fig. 3A). SEM images of *E. faecalis* inoculated in the QPEI nanoparticle suspension revealed numerous nanoparticles in close proximity to the *E. faecalis* cell walls; nanoparticles were not seen in the control group (Figs. 3B and C, respectively).

**Figure 4:**
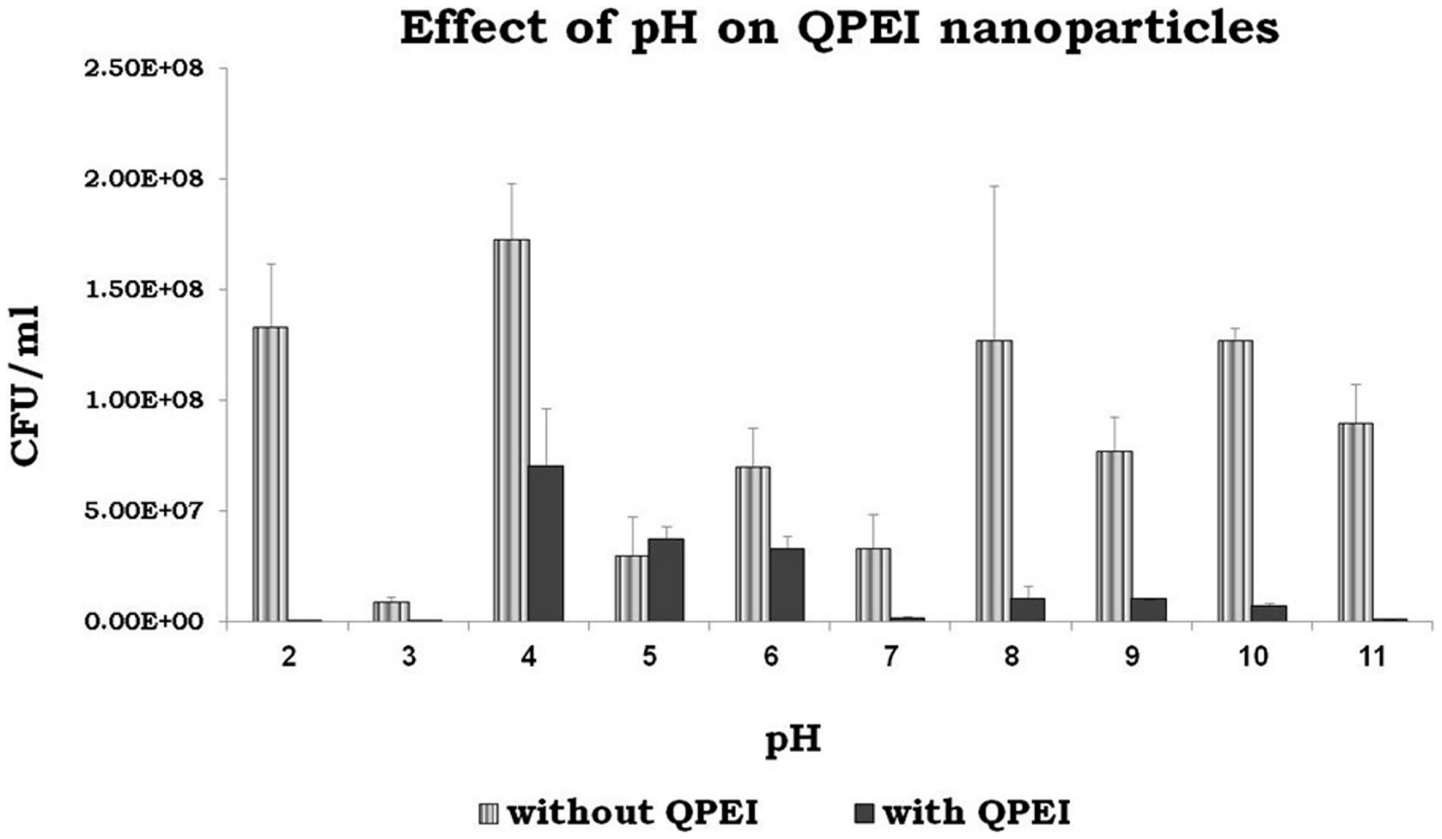
Determination of the antibacterial activity of QPEI nanoparticles at different pH values. *E. faecalis* viable counts determined after 10 min incubation in DDW with and without QPEI nanoparticles at various pH values. Serial dilutions were made to determine the viable cell counts calculated as CFU/mL. The antibacterial ratio (growth inhibition) was calculated according to the equation: no. of original cells - no. of viable cells/ no. of original cells x 100. The antibacterial activity of the nanoparticles tested at different pH values showed a viable count reduction at pH values >5 and <5. At pH 5.0, the viable counts were similar to those of the control group.

The antibacterial activity of the nanoparticles tested at different pH values showed a reduction in viable count at pH values >5.0 and <5.0. At pH 5.0 the viable counts were similar to those of the control group (Fig. 4).

### Cytotoxicity assay

The *in situ* manual incorporation of nano-sized quaternary amine particles in an epoxy- based endodontic sealer was found to be non-cytotoxic (table 2). Cells treated with the negative control showed no decolorization or cell lysis, i.e. there was no cell reaction (0/0). The positive control showed a median cell lysis of 5, resulting in a cell reaction of 2-5, interpreted as moderately-severely cytotoxic. The novel endodontic sealer showed a median decolorization zone of 0 and median cell lysis of 0 (0/0), interpreted as non-cytotoxic.

**Table 2:** Cytotoxicity of the novel endodontic sealer.

Treatment	Well no.	Decolorization index	Cell lysis index	Cell reaction	Scale	Interpretation of cytotoxicity
**Negative control**	**1**	**0**	**0**	**0/0**	**0**	**Non cytotoxicity**
	**2**	**0**	**0**	**0/0**	**0**	
	**3**	**0**	**0**	**0/0**	**0**	
	**Median**	**0**	**0**	**0/0**	**0**	
**Positive control**	**1**	**2**	**5**	**2/5**	**2**–**3**	**Moderately- Severely cytotoxic**
	**2**	**2**	**5**	**2/5**	**2**–**3**	
	**3**	**1**	**5**	**2/5**	**2**–**3**	
	**Median**	**2**	**5**	**2/5**	**2**–**3**	
**RCS + 1.5% QPEI nanoparticles**	**1**	**0**	**0**	**0/0**	**0**	**Non- cytotoxic**
	**2**	**0**	**0**	**0/0**	**0**	
	**3**	**0**	**0**	**0/0**	**0**	
	**Median**	**0**	**0**	**0/0**	**0**	

Decolorization and Lysis Indices of L929 cell cultures treated with the negative control - high density polyethylene film; positive control – ZEDC; and the test products - RCS + 1.5% (wt/wt) QPEI nanoparticles. The median was calculated for three replicates from each test group (1 per well). The cell reaction (decolorization index/lysis index), scale and interpretation are presented for each treatment. The results show that the novel endodontic sealer is non-cytotoxic.

### Physical properties

Further tests of the novel endodontic sealer showed that incorporation of the QPEI nanoparticles did not have a negative effect on the sealer's physical properties, such as flow and solubility compared with those of the conventional sealer (table 3). Measurement of the glass transition temperature (Tg) showed an acceptable minimal difference for all the test groups: 0%, 1%, 1.5% or 2% (wt/wt) QPEI nanoparticles.

**Table 3:** Physical properties of sealer incorporating QPEI nanoparticles.

(%) nanoparticles in RCS	0	1	1.5	2
a **Solubility (%)**	**0.023**	**0.021**	**0.029**	**0.030**
b **Tg (midpoint) (°C)**	**30.8**	**29.9**	**29.9**	**27.5**
**^c^ Flow (cm)**	**Pass (2.9)**	**Pass (2.8)**	**Pass (2.6)**	**Pass (2.1)**

a Solubility tests were performed in accordance with ISO 6876:2001(E) specifications for root canal sealing materials. Samples were weighed three times on an HM-200 precision scale. The mean reading was then recorded. All solubility results were in the clinically accepted range, in accordance with ISO specifications.

b Thermal analysis during gradual heating (0°C – 150°C; 10°C /min) of RCS samples incorporating 0%, 1%, 1.5% or 2% QPEI nanoparticles. Analysis of all samples was performed using a differential scanning calorimeter (DSC) device. Samples were cooled to 0°C and then heated to 150°C at a heating rate of 10°C/min. Thermal transition values were calculated using STAR software. All thermal transitions were in the same clinically accepted range.

C Flow tests were conducted according to ISO 6876:2001(E) specifications. Three determinations were made and the mean value was calculated to the nearest millimeter. The minimal acceptable diameter was 20 mm. The results of all the test groups were in the clinically accepted range, in accordance with ISO specifications.

## Discussion

This study is the first to provide an overview of the clinical potential of a novel endodontic sealer. The novel sealer affixes polycationic nanoparticles, achieving surface bactericidal properties, but at the same time impeding nanoparticle penetration into eukaryotic cells and thereby mitigating a possible toxic effect. Furthermore, the antibacterial potency was evident when < 2% (wt/wt) nanoparticles were incorporated into the endodontic sealer, maintaining adequate physical properties. Our results show that this novel endodontic sealer exhibits a potent and prolonged antibacterial activity, and is of potential therapeutic use. Some of the endodontic sealers allegedly contain antimicrobial components such as eugenol and zinc oxide, silver,

hexamethylenetetramine, calcium oxide, calcium hydroxide, and epoxide resin [35]–[38]. For the sealer to be effective against microorganisms, these compounds need to be released from the matrix into the surrounding milieu. However, it has been shown that the antibacterial effect is of short-term nature, evident in the freshly mixed sealers, and is greatly reduced after material setting [39]. The upside of this phenomenon is that these compounds, which may have a toxic effect on the surrounding tissues, become less harmful when their concentration is reduced.

In the present study the antibacterial effect was tested against a clinical isolate of *E. faecalis*. This microorganism can be detected in primary endodontic infections and is frequently recovered from treatment failures. Furthermore, the average odds of detecting *E. faecalis* in cases of persistent infections associated with treatment failure were reported to be 9.1 [40]. This bacterium plays a critical role in endodontic pathology; *E. faecalis* was found in 60% of teeth with chronic apical periodontitis and in 72% of teeth with secondary infection [41]. Given the significance of preventing endodontic infection in the success of root canal therapy, the antimicrobial effect of the novel sealer was tested against *E. faecalis*, with the end goal of eliminating residual microorganisms that might be resistant to both chemo-mechanical preparation and intra-canal medication. Our results show that the novel sealer has a significant antibacterial effect against *E. faecalis* that come in direct contact with the material's surface, as can be seen in SEM micrographs and in the DCT results and viable cell counts. In addition, we tested the antibacterial effect of the novel sealer, using the ADT diffusion test. The rationale for the test was that it has been used extensively to assess the antibacterial activity of dental materials. Two major shortcomings of this method include its inability to differentiate between the bacteriostatic or bactericidal properties of the material tested, and that the antibacterial effect is greatly influenced by the diffusion ability of the material across the agar, a feature that most dental materials lack as a rule [42]. No antibacterial halo was seen around the novel sealer or the control sealer. In addition, to eliminate the possibility that the antibacterial activity was due to bioactive components released from the set sealers, the antibacterial effect of the medium in which the sealers were immersed was tested. The results showed that the bacterial outgrowth was similar to that seen in the control elute. That finding and as materials which diffuse more easily will probably provide larger zones of inhibition in ADT, it may be speculated that both the novel sealer and the control sealer did not release antibacterial compounds after setting. This indicates that the antibacterial activity of the novel sealer observed in the other antibacterial tests can be attributed to direct contact with the materials. In the DCT, which is a quantitative method, the O.D. represents a reliable indirect method of testing the viability of the bacteria. As shown, a rise in O.D. values in the DCT correlated with the bacterial growth, according to the viable bacterial counts. Quantification of the results is possible using DCT by including a set of growth curves as a standard in each plate, with serial dilutions, allowing exact calculation of the viable bacteria (CFU/ml) following direct contact with the tested materials' surface. Using the DCT most of these variables can be carefully controlled, allowing consistent and reproducible results. In this test a significant decrease of 6 logs was recorded in the viable count of bacteria that came in contact with the novel sealer incorporating QPEI nanoparticles. These results may indicate that the novel sealer is likely to have antimicrobial activity within the root canal system. When testing the effect of suspended QPEI nanoparticles on bacteria, minimal antibacterial activity was recorded at pH 5.0, which may indicate the isoelectric point of the.*E. faecalis* proteins, as suggested by *Gao et. al*. [29]. Thus, it can be speculated that at this isoelectric point, when the electrostatic attraction between the nanoparticles and the bacterial membrane disappears, the antibacterial effect of the QPEI nanoparticles is minimal. Above this point, the cells become negatively charged and the electrostatic interaction between the QPEI nanoparticles and the cells increases, resulting in a greater antibacterial effect. Although it is logical that at pH < 5.0 the antibacterial effect should be weakened due to less interaction between the nanoparticles and the cells, the results show an increased effect. A possible explanation for the antibacterial activity at very low pH levels is that the main antibacterial effect comes from the H^+^ ions, with a minimal contribution from the nanoparticles themselves.

The novel sealer presented contained the incorporated quaternary ammonium nanoparticles, which have a positive surface charge and are hydrophobic. Their bactericidal mechanism is not fully understood. It was suggested that ammonium compounds are absorbed and penetrate through the bacterial cell wall. These nanoparticles merge with the protein layer and analogous lipid fat layer and subsequently block the normal exchange of ions and substances, leading to leakage of intracellular contents and cell death [29]. Although a toxic effect against microorganisms is an advantageous quality, sealers are expected to be biocompatible with the surrounding tissues. Regrettably, it has been reported that most sealers pose significant cytotoxic risks, their cytotoxicity generally increasing with time [43]. Our results show that both the control sealer and the novel sealer posed no significant cytotoxic risk and emphasize the need to develop sealers that encompass sealing ability, antimicrobial properties and biocompatibility.

Endodontic sealers that possess both optimal flow ability and optimal antimicrobial properties may theoretically assist in the elimination of microorganisms located in confined areas of the root canal system [44]. Generally, filling techniques for root canals include core obturating materials, such as gutta-percha, and endodontic sealers that fill canal irregularities and minor discrepancies between the root canal wall and the core material. Consequently, root sealers may enhance impermeability if they have adequate flow ability. Bearing this in mind, the flow ability of the novel sealer was tested. Although the novel sealer has lower flow ability than that of the control sealer, the difference was clinically irrelevant, as it was within the acceptable ISO range. Moreover, sealers that possess both favorable flow ability and antimicrobial properties might theoretically eradicate microorganisms located in confined areas of the root canal system.

Additional properties such as glass transition temperature (Tg) and solubility are required for the proper function of an endodontic sealer. Tg is commonly used to monitor the degree of conversion (DC) and morphology of cross-linked polymers in general and particularly for epoxy resin-based materials such as endodontic sealers. Tg values rise as the curing reaction is propagated and the three dimensional network of the monomers is being formed. The presence of inert plasticizers like silicone oil, un-reacted monomers or thermally unstable polymers will lead to a decrease in Tg. In some cases, when long chains of polymer form, rather than a branched network, melting at temperatures higher than the Tg is likely. Tg and DC values measured in the novel sealer were not significantly different from those obtained for the control sealer. As Tg is an informative parameter regarding the degree of monomer conversion for epoxy resins, it can be concluded that the addition of QPEI nanoparticles at up to 1.5% does not affect the setting of the RCS material. The addition of 2% QPEI nanoparticles slightly reduces Tg. This may be the result of the lower Tg of the nanoparticles rather than inhibition of the RCS setting process. Incomplete polymerization and a post curing reaction were observed in all the samples, regardless of nanoparticle affixation. Additional sealer characteristics that may influence the success of root canal treatment include low solubility.

## Conclusions

In conclusion, sealing ability, biocompatibility, and antimicrobial activity are critical characteristics essential for the success of root canal treatment. In the present *in vitro* study a novel sealer encompassing such features was introduced. It seems reasonable to presume that the use of an endodontic sealer that has both antimicrobial activity and a good flow rate may bolster efficient microbial control within the root canal system. Furthermore, the hazardous spread of bacterial resistance to conventional antibiotics requires the use of non-specific antimicrobial means whenever possible. As shown in the present study, affixation of polycationic antimicrobial nanoparticles in an endodontic sealer revealed long-lasting antimicrobial potency, providing an effective antimicrobial alternative. Nonetheless, it should be pointed out that since *in vitro* studies have clear limitations, clinical presumptions must be made with the utmost prudence. Further *in vitro* studies should be focused on the antibacterial efficacy of this novel endodontic sealer when recontamination of the root canal occurs after saliva challenge.
